# Respiratory viral infections in children with asthma: do they matter and can we prevent them?

**DOI:** 10.1186/1471-2431-12-147

**Published:** 2012-09-13

**Authors:** Hamid Ahanchian, Carmen M Jones, Yueh-sheng Chen, Peter D Sly

**Affiliations:** 1 The Queensland Children’s Medical Research Institute, The University of Queensland, Brisbane, Australia; 2 Allergy research Center, Mashhad University of Medical Sciences, Mashhad, Iran

**Keywords:** Acute respiratory infections, Childhood asthma, Common cold, Acute exacerbations, Rhinovirus

## Abstract

**Background:**

Asthma is a major public health problem with a huge social and economic burden affecting 300 million people worldwide. Viral respiratory infections are the major cause of acute asthma exacerbations and may contribute to asthma inception in high risk young children with susceptible genetic background. Acute exacerbations are associated with decreased lung growth or accelerated loss of lung function and, as such, add substantially to both the cost and morbidity associated with asthma.

**Discussion:**

While the importance of preventing viral infection is well established, preventive strategies have not been well explored. Good personal hygiene, hand-washing and avoidance of cigarette smoke are likely to reduce respiratory viral infections. Eating a healthy balanced diet, active probiotic supplements and bacterial-derived products, such as OM-85, may reduce recurrent infections in susceptible children. There are no practical anti-viral therapies currently available that are suitable for widespread use.

**Summary:**

Hand hygiene is the best measure to prevent the common cold. A healthy balanced diet, active probiotic supplements and immunostimulant OM-85 may reduce recurrent infections in asthmatic children.

## Background

Asthma is a major public health problem with a huge social and economic burden affecting 300 million people worldwide [1]. Viral respiratory infections are the major cause of acute asthma exacerbations and contribute to asthma inception in high risk young children with susceptible genetic background. A history of wheeze associated with respiratory viral infections early in life is one of the major risk factors for the later development of asthma [2-7], together with sensitization to aeroallergens in early life and a family history of asthma and allergies, reflecting a genetic predisposition. Respiratory viral infections are also the principal cause of asthma exacerbations in children and adults [8-13]. However, the question of whether viral infections “select” susceptible hosts or whether viral infections may induce asthma * de novo * by “damaging” airways is not settled. In other words, do viruses cause or simply unmask asthma?

### Viral infections and innate immune responses

Respiratory viruses first infect nasal epithelial cells which triggers an antiviral response. This response is driven by type I (α/β) and III (λ) interferons (IFN) that are induced following recognition of viral ribonucleic acid (RNA) by pattern recognition receptors (PRRs). Toll-like receptors (TLRs) are cell surface and endosomal PRRs, whilst the RNA helicase receptors (RIG-I and MDA-5) and NOD-like receptors (NOD2), detect viral RNA in the cytoplasm. Signalling via the PRRs activates transcription factors (IRF-3, IRF-7, NF-κB), which lead to the production and secretion of type I and III IFN. The IFNs then bind to cell surface receptors to activate a separate pathway leading to the production of interferon stimulated genes (ISGs) which encode antiviral proteins that combat infection, as well as PRRs and transcriptional factors which further amplify IFN production. The respiratory syncytial virus (RSV), human meta-pneumovirus (hMPV) and human rhinovirus (HRV) are all single stranded RNA viruses but engage differently with cell signalling pathways. In airway epithelial cells RSV and hMPV RNA are primarily detected by RIG-I in the cytoplasm [14,15]. RSV can also be detected by NOD2 [16]. HRV is endocytosed by epithelial cells, and is therefore primarily detected by TLR3 in the endosome early in the infection process and by RIG-I and MDA-5 later in infection following upregulation of these PRRs [17]. The fusion (F) protein of RSV is recognised by TLR4 at the epithelial cell surface [18].

A successful antiviral response would see the infection limited to the upper airway, as is the case clinically with the majority of viral infections in healthy individuals. Should such a response be deficient, then predominantly upper-airway viral infections, such as HRV, may spread to the lower airways, causing lower respiratory symptoms and an exacerbation of asthma in predisposed individuals.

### Abnormal innate antiviral immunity in asthmatics

While definitive data are yet to be produced, experimental HRV infections in adult volunteers initially suggested that asthmatics were more likely to develop lower respiratory infections (LRI) than healthy adults, i.e. less likely to be able to limit viral replication to the upper airways [19,20]. Subsequent * in vitro * infection of primary airway epithelial cells from asthmatic and healthy adults with HRV have demonstrated that asthmatic cells produce less IFN-β [21] and IFN-λ [22] making them potentially more susceptible to infection, slower to clear infection, and more susceptible to virus-induced cell cytotoxicity. Deficiencies in the IFN-α response of peripheral blood mononuclear cells and plasmacytoid dendritic cells from asthmatic adults and children has also been observed, in these particular studies, in response to RSV, HRV [14,15] and Influenza A [23]. It is likely that the overall impaired innate immune response of the asthmatic airway epithelium is a result of deficiencies in the antiviral response of both epithelial cells and immune cells. Childhood, especially infancy, is characterized by developmentally-regulated deficiencies in innate and adaptive immunity [24]. Such deficiencies are likely to increase the risk of viral LRI in children, especially in those at high risk for asthma and allergies.

### Viral infections in children with asthma

Each year, at the end of summer, parents of asthmatic children are concerned about acute asthma exacerbations following a common cold, asking how to minimize the risk during the winter viral season. It is a valid concern as up to 70% of asthmatic children have an intermittent or wheeze which is mostly symptomatic after viral infections [25,26]. Asthmatics with exacerbation-prone phenotype are susceptible to acute exacerbations requiring hospitalization or an unscheduled visit for medical attention. Major risk factors for acute exacerbations include previous acute exacerbation, allergy, young age, poorly controlled asthma, and, in particular, viral respiratory infections. Moreover, recent data suggests an interaction between allergies and viral infections occurs to increase the risk of asthma exacerbation [27]. Acute exacerbations are associated with decreased lung growth or accelerated loss of lung function and, as such, add substantially to both the cost and morbidity associated with asthma [28,29]. Viral respiratory infections are the main cause of asthma exacerbations in children (80-85%) and are a major risk factor for admission in hospital every autumn [30-32]. HRV are the most common viral agents [33]; Other respiratory tract viruses detected in children with an asthma exacerbation include RSV, influenza, coronavirus, hMPV, parainfluenza virus, adenovirus, and bocavirus [34-36]. Current drugs for the prevention and treatment of virus-induced exacerbation of asthma are poorly effective and novel alternative therapies are needed.

### Role of respiratory viral infections in asthma inception

Much research interest has focused on the potential role respiratory viral infections play in the inception of asthma. It is well established that hospitalization for RSV bronchiolitis is a risk factor for asthma during childhood [37,38]. Epidemiological studies have shown an increased risk of asthma with LRI caused by HRV. In the Childhood Origin of Asthma (COAST) birth cohort study, wheezing with RSV (odds ratio [OR], 2.6), HRV (OR, 9.8), or both HRV and RSV (OR, 10) was associated with increased asthma risk at age six years [7]. The Childhood Asthma Study (CAS) in Perth, Australia showed that wheezing with HRV or RSV in the first year of life was a risk factor (OR, 2.5) for current wheeze at five years of age [4]. Infant birth about four months before the winter virus peak carried the highest risk of developing asthma compared with birth 12 months before the peak [39]. The risk of asthma is increased by severe LRI (sLRI), especially in the presence of allergic sensitization in early life [4,25]. There appears to be a synergistic interaction between viral infection and allergic sensitization, suggesting a “two hit” model for induction of persistent asthma. These data also provide a series of novel strategies for the primary prevention of asthma by prevention of either allergic sensitization or of sLRI in high risk children. This strategy is also supported in a study by Simoes et al. [40], in which the use of palivizumab to prevent RSV infection decreased the risk of recurrent wheezing in nonatopic premature infants.

The crucial period, with respect to asthma initiation, appears to be the first two to three years of life during which the growth and remodelling of lung and airways proceeds at maximum rates. Pulmonary inflammation resulting from atopy and sLRI occurring during this vulnerable time is hypothesized to perturb underlying tissue differentiation programs, resulting in deleterious long term effects on respiratory functions. As a result, there is widespread belief amongst the paediatric respiratory community that intervention measures that can lower the frequency and/or intensity of sLRI in early life amongst the high risk atopic subgroup of children are likely to be successful at preventing asthma. If successful, these strategies would have major implications for reducing the high impact of this chronic disease on the community [17,41,42].

Recent studies using culture-independent techniques have challenged the long-held dogma that lungs are sterile and have demonstrated that a microbiota community exists in the lung [43-45]. The implications of these new data are not clear, however new concepts and more research is required. The resident microbiome is different in the presence of respiratory disease [45,46]; therefore interactions between respiratory viruses and the resident pulmonary microbiome are postulated. The pulmonary and gastrointestinal microbiota influence the immune system and interventional approaches (by bacterial immunostimulants, prebiotics and/or probiotics) to create a healthy gut and respiratory microbiota are potential strategies for the prevention of viral infections [45].

### Preventing viral infections by non-immunologic methods

Children are important vectors for HRV transmission to family members particularly siblings [47,48]. HRV shedding peaks two to four days after infection and decreases sharply thereafter, although nasal samples can be positive for rhinovirus for up to five weeks after a symptomatic infection [49].

There are three ways of common cold transmission in children. First, inhalation of small particles aerosolized by coughing; second, large particle droplets from saliva expelled while sneezing; and third, self-inoculation of one’s own conjunctivae or nasal mucosa after touching a person or object contaminated with the cold viruses. The first two methods are inefficient [50], while the third is the most important method of transmission. The mode of transmission could differ with age of the index case, duration of contact, and other factors. Moreover, there is some evidence that the daily activities of infected people can lead to the contamination of environmental surfaces with HRV e.g. light switches, telephone dial buttons and handsets [48].

Meticulous hand hygiene is the best measure to prevent the common cold; frequent hand washing and avoid touching one's nose and eyes [51-53]. The use of alcohol-based hand sanitizers is also effective [54,55]. The promotion of handwashing was associated with a 12-34% reduction in respiratory-tract infections and colds in child-care centres in the USA [56] Canada [57] and Australia [58] and a 21% decrease in absences due to respiratory illness in the school setting [56]. Hand hygiene campaigns were also successful in reducing absenteeism caused by influenza-like illnesses among schoolchildren in Egypt [59]. Similar programs within families would be expected to reduce transmission of HRV between family members.

A recent Cochrane review which included data from 67 randomised controlled trials and observational studies, investigated the effectiveness of physical interventions to reduce the spread of respiratory viruses. The authors concluded that respiratory virus spread can be reduced by hygiene measures (such as handwashing), especially around younger children and can reduce transmission from children to other family members [51]. Controversy still exists and a newly published study showed that an antiviral hand treatment used by adult volunteers, recruited from a university community, did not significantly reduce RV infection or RV-related common cold illnesses [60].

Asthmatic children should avoid close contact with people who have colds especially during the first three days of their illness. There is little evidence to support the effectiveness of face masks to reduce the risk of viral respiratory infections and consequently, the use of mask is generally not recommended for prevention of common cold [51,61].

### General immunologic strategies

Immune function and anti-viral defenses have a number of components, both specific and non-specific. Asthmatic children can improve their immune function by following some simple advice including a healthy life style with regular exercise, a balanced diet, adequate sleep and avoiding environmental tobacco smoke, stress and unnecessary antibiotics.

### Exercise

Exercise has anti-inflammatory effects and in the long term can protect the development of chronic diseases and obesity [62]. Regular exercise of moderate-intensity is associated with a reduced incidence of upper respiratory tract infection. However, long hours of intensive training appear to make children more susceptible to upper respiratory tract infections [63-65]. The recommended means of aerobic exercise is walking, with an optimal frequency of three to five days a week and an optimal duration of 20 to 30 minutes of continuous activity [66]. In a recent study, the IgA secretion rate was negatively correlated with the incidence of infections [67]. A recent randomized trial comparing meditation and exercise with wait-list control among adults aged 50 years and older found significant reductions in ARI illness [68].

### Diet

Malnutrition is the most common cause of immune deficiency worldwide and a balanced diet is fundamental for a healthy immune system. Vitamin D deficiency has been associated with increased risk of infections, early-life wheeze and reduced asthma control [69,70]. Vitamin A derivatives are involved in the regulation of the immune system and tissue inflammation as well as prevention of respiratory infections [71]. Zinc, selenium and other trace elements are necessary for function of both innate and adaptive immune function. A high intake of fruit and vegetables ensures adequate consumption of nutrients and antioxidants and appears to be beneficial for asthma. Although recent reviews have shown that zinc [72], garlic [73], Echinacea purpurea [74] or Ginseng [75] supplementation for several months may reduce cold incidence, there is insufficient evidence to recommend any vitamin or mineral supplementation in the management of asthmatic children without nutrient deficiency [76,77]. However, a large controlled trial showed Echinacea was ineffective in reducing infection rate or symptom severity of HRV infection in healthy young adult volunteers [78]. Vitamin C supplementation failed to reduce the incidence of colds in the general population except in those exposed to short periods of extreme physical stress [79]. Finally, it is worth remembering that infants who are not breastfed have significantly higher risk of respiratory, gastrointestinal, and other infections, as breast milk is a biologically active substance containing antimicrobial and immunomodulatory elements [80-82].

### Sleep

Sleep and the circadian system exert a regulatory influence on immune functions. Sleep deprivation can affect immune function in several ways including reduced natural killer cell activity, suppressed interleukin-2 production and increased levels of circulating proinflammatory cytokines [83,84]. There is also evidence for an enhanced susceptibility to the common cold and pneumonia with poor sleep efficiency [85,86].

### Air pollution

Air pollutants (nitrogen dioxide, ozone, particulate matter) and environmental tobacco smoke (ETS) have long been correlated with multiple adverse effects on the immune system and susceptibility to viral respiratory tract infections in children [87-90]. Studies in Europe and the United States have shown that 40% of children live with a smoker [34] and they have approximately twice the risk of contracting a serious respiratory tract infection in early life [91]. Cigarette smoking leads to a longer duration of cough, greater frequency of abnormal auscultatory findings during acute respiratory tract illness [92,93] and higher risk for severe exacerbations [94]. Urinary leukotriene E4 levels identify children exposed to ETS at high risk for asthma exacerbation [94].

### Specific immunologic strategies

There is strong evidence that some pharmacological preparations can help prevent viral infection by specific effects on immune system. These results have been promising with a hope that using these strategies can attenuate the role of viruses in asthma inception.

### Probiotics and prebiotics

Ancient physicians of the Middle East prescribed yogurt for curing disorders of the stomach, intestines and for stimulation of appetite. It is written in the old Persian Testament that “Abraham owed his longevity to the consumption of sour milk” [95]. The popularity of probiotics and intestinal microbiota significantly increased when the Nobel Prize-winning Russian scientist Eli Metchnikoff suggested in 1908 that the long life of Bulgarian peasants resulted from their consumption of fermented milk products [96]. The term probiotic, meaning for life, is used for live micro-organisms (typically of the bifidobacterium and lactobacillus species) administered in adequate amounts which confer a beneficial physiological effect on the host. Prebiotics are nutrients, in particular oligosaccharides, which foster the growth of probiotics in the colon. The term synbiotics is used when a product contains both probiotics and prebiotics [97].

Up to 100 trillion bacteria from different species colonize the human gut [98]. This microbiota participates in: host metabolism, vitamin synthesis, control of epithelial cell growth, protection from infectious microbes, and helps proper development and function of the immune system. There is constant cross-talk between microbiota and gut-associated lymphoid tissue (the largest lymphoid tissue of the human body which contains more than 60% of all body lymphocytes) to establish mucosal immune tolerance in the gut. Common mucosal immunity describes the phenomenon where immune cells, especially regulatory T-cells, traffic to and influence responses at other mucosal surfaces, including the lungs [99]. Alteration in the microbiota composition(dysbiosis) results in immunological dysregulation that may underlie many human diseases such as inflammatory diseases [100], obesity [101], allergy [102] and autoimmunity [103].

Reduced bacterial diversity in the infant’s gastrointestinal tract has been associated with an increased risk of allergic sensitization and allergic rhinitis but not asthma or atopic dermatitis [102]. In the first year of life, especially the first few weeks, the microbiota of the newborn is highly variable during this critical time of post-natal maturation of the immune system. Microbiota is shaped by genetic and environmental factors including: mode of delivery, neonates born by means of vaginal delivery are exposed to mothers gut, skin, and vaginal flora [104]; breast feeding and diet [105]; farm or urban living [106]; vitamin D status [107]; and antibiotic consumption [98,108]. This knowledge stimulated interest in the use of probiotics and prebiotics as the intentional introduction or encouragement of specific microbes to shape immune system development. Specifically, the microbiota can activate distinct tolerogenic dendritic cells in the gut and through this interaction can drive regulatory T-cell differentiation that modulates both Th1 and Th2 responses inside and outside the gut [109-111]. Probiotics have been successfully used for the treatment of several gastrointestinal disorders (viral and antibiotic-associated diarrhea, inflammatory bowel disease) [112,113]. However, attempts to prevent or treat allergic disorders such as eczema, asthma and allergic rhinitis have had inconsistent results [99,109,114-116].

There are a growing number of clinical trials using probiotics for the prevention and management of respiratory infections. While the precise mechanisms are largely unknown, speculations include: probiotics compete against pathogens; increase the barrier function in respiratory epithelium; immunostimulatory effects by enhancing cellular immunity with increased activity of natural killer cells and macrophages in airways [117]. Probiotics reduce the frequency of gastrointestinal and respiratory tract infections in children who attend day care centres [118]. They have also been found to reduce the incidence of ventilator-associated pneumonia, respiratory infections in healthy and hospitalized children, and reduce the duration of common cold symptoms [119-122].

One study demonstrated that that daily probiotic supplementation for six winter months in children three to five years of age reduced the incidence of fever, coughing and rhinorrhea by 32-43% with no notable adverse events [123]. Probiotic combination with vitamins and minerals also reduced the duration and severity of common cold [124]. A recent Cochrane review of 14 randomised controlled trials showed that probiotics were better than placebo in reducing the number of episodes of acute upper respiratory infections (URIs) and reducing antibiotic use, while there were no differences in the mean duration of an episode and no increase in adverse events [125]. Probiotic foods such as probiotic milk or yogurt (functional foods) containing well-defined probiotic strains may reduce the risk of catching the common cold and represent a simple, safe, effective, available and affordable method for preventing respiratory infections in children [112,120,126-131].

Although there are several clinical trials that showed the preventive effect of probiotic, prebiotic [132] or synbiotics treatments [133] on respiratory infections, not all studies are positive with some failing to show any significant preventive effect [134]. To explain the different results in clinical trials, it is of particular importance to point out that the immunomodulatory capabilities of probiotics are strain-dependent. Difference in dosage, duration of intervention, population and environmental background may also affect the results. One major limitation in this field is that it is not possible to test just how “probiotic” a particular preparation is. Technical advances will be required before some of the apparent discrepant results of studies can be resolved.

### Immunostimulants

Several immunostimulants, including herbal extracts, bacterial extracts, synthetic compounds, have been promoted as increasing the immune defences of the respiratory tract. A recent Cochrane review included data from 35 placebo-controlled trials including 4060 participants below the age of 18 years in which various types of “immunostimulants” were used to reduce acute respiratory tract infections, involving either upper or lower airways. The authors concluded that immunostimulants reduced the incidence of acute respiratory infections by 40% on average in susceptible children, but that trial quality was generally poor and a “high level of statistical heterogeneity was evident”. A subgroup analysis focusing on bacterial immunostimulants, including OM85, produced similar results with lower statistical heterogeneity [135].

OM-85 BV (Broncho-Vaxom) is an immunostimulant extracted from eight common bacterial pathogens of the upper respiratory tract: * Haemophilus influenzae, Diplococcus pneumoniae, Klebsiella pneumoniae and ozaenae, Staphylococcus aureus, Streptococcus pyogenes and viridans *, * Neisseria catarrhalis * and has been used in several countries around the world for as long as 20 years [136]. Recent studies showed that OM-85 BV can reduce the number of acute respiratory infections by 25% to 50% compared with placebo in children with a history of recurrent infection [137]. Of particular interest, Razi et al. showed that children between the age one and six years with recurrent wheezing who were given OM-85 BV had a 40% reduction in the rates of wheezing over the subsequent 12 months, compared to placebo (p < 0.001). In addition, the duration of each wheezing attack was two days shorter in the group given OM-85 BV than in the group given placebo (* p * = 0.001) [138].

Again, direct evidence of the mechanisms involved are lacking from human studies. However, recent data from rodents shows that baseline regulatory T lymphocyte activity in the airways can be boosted by microbe-derived stimulation of the gut [139]. Bacterial immunostimulants were also shown to enhance innate immunity (i.e. intensification of phagocytosis) and adaptive immunity [140].

### Interferons

As discussed above, evidence exists for an impaired innate immune response to respiratory viral infections in asthmatics [141]. Entry of rhinovirus into normal epithelial cells initiates a vigorous innate immune response with IFN-β secretion and apoptosis induction. In asthma, IFN-β and IFN-λ responses are impaired, resulting in viral replication, cell cytotoxicity, enhanced virion shedding and increased susceptibility to common cold [17,142]. Epithelial cells of asthmatic patients responded to exogenous treatment with IFN-β exhibiting reduced rhinovirus release (Cakebread, Xu et al. 2011; Jackson, Sykes et al. 2011). If the proposed deficiency of type I and III contribute to asthma exacerbations [21,22,143], correcting this deficiency with exogenous interferons would be a logical approach. The advantages of interferon application include the broad spectrum of activity with low risk of resistance development [47]. Prophylactic intranasal recombinant IFN-α and IFN-β have been shown to be effective against rhinovirus infection in humans [144-146]. The results of these clinical trials are awaited with interest [147,148]. However, the systemic symptoms associated with severe viral infections, e.g. influenza, are associated with interferons, so careful dosing may be required. Considering the occurrence of the local side effects, neutropenia and cost, the use of long-term prophylaxis with daily, intranasal administration of interferons is not feasible [144]. However, randomized clinical trials using similar strategies are currently underway in adults with chronic respiratory disease and the results are keenly awaited.

### Vitamin D

Vitamin D deficiency is a common worldwide problem [149,150]. Beside importance for bone health, vitamin D plays an important role in adequate function of both the innate and adaptive immune systems including development of dendritic cells and regulatory T lymphocytes [151,152] production of antimicrobial proteins by airway epithelium [153], modifying the effect of intestinal flora on inflammatory disorders [107], and modulation of the inflammatory response to viral infections [154]. Recent reports suggest that vitamin D might play a role in the recent increase in allergic disease [155-157]. Vitamin D insufficiency has been associated with a higher incidence of respiratory tract infection, wheezing illness in children [158], reduced asthma control [159], emergency department visits, severe asthma exacerbations and hospitalizations [70,160]. In a recent study of 48 children from five to 18 years of age, with newly diagnosed asthma, vitamin D supplementation during the northern hemisphere winter months (September to July) prevented declining serum concentrations of 25(OH) D and reduced the risk of asthma exacerbation triggered by acute respiratory tract infections [161].

### Macrolides

Macrolides possess anti-inflammatory and immunomodulatory properties extending beyond their antibacterial activity [162]. Indeed, they can attenuate pro-inflammatory cytokine production by bronchial epithelial cells, neutrophils and macrophages that may contribute to clinical improvement in many patients with chronic airway inflammation [163-165]. Azithromycin has anti-rhinoviral activity and can reduce HRV replication and release by increasing interferon production from epithelial cells [42,166,167]. Macrolide antibiotics inhibit RSV infection in human airway epithelial cells [168]. A three weeks treatment with clarithromycin in RSV bronchiolitis had statistically significant effects on hospital length of stay and rate of readmission to the hospital within six months after discharge [169]. However, direct evidence of macrolides preventing respiratory viral infection in children is lacking.

### Anti-viral therapies

As the majority of respiratory viral infections in young children are caused by HRV or RSV, we will briefly discuss anti-viral strategies to prevent HRV or RSV infections in asthmatic children. Because there are more than 100 serotypes of HRV, antiviral drugs are considered to be more effective than vaccination. Antiviral agents have been designed to inhibit rhinovirus attachment, entry to the cell, viral uncoating, and RNA and protein synthesis [47]. Table 1 shows how intervention strategies can be targeted to various steps in the infective process. 

**Table 1:** The processes of rhinoviral infection and preventive strategies

**Process**	**Preventive strategies**
**Rhinoviral transmission**	Hand hygiene, isolation
**Attachment to respiratory epithelium**	HRV neutralizing antibodies, anti-receptor antibodies
	Second generation antihistamines, zinc vaccines
**Entry, RNA and protein synthesis**	Anti-rhinoviral therapies (Pleconaril, Ruprintrivir)
**Enhancing immunity**	Balanced diet, interferons, immunostimulants, probiotics, breast milk, Echinacea, garlic, zinc, ginseng

### Rhinovirus structure

HRV has the icosahedrally shaped capsid formed by 60 identical copies of viral capsid structural proteins VP1-4. The capsid protects the single-stranded, positive sense RNA genome. While HRV-A and -B most often induce a self-limited upper respiratory infection, the recently discovered HRV-C was associated with sLRIs in infants, bronchiolitis, and asthma exacerbations in children [170,171].

### Prevention of attachment, entry and uncoating

HRV deposits on nasal or conjunctival mucosa and is transported to the posterior nasopharynx by mucociliary action of epithelial cells [172]. The so-called major group of HRV uses intercellular adhesion molecule-1 (ICAM-1) as their receptor [173] and the minor group attach to low density lipoprotein (LDL) receptor and very-LDL (VLDL) receptors on epithelial cells in the adenoid area to bind and enter cells [174,175]. Viral attachment can be prevented by specific anti-HRV neutralizing antibodies, anti-receptor antibodies and soluble receptor molecules.

Endothelial cells express histamine receptors and increased adhesion molecule expression, such as ICAM-1, was demonstrated by histamine infusion. Second-generation H1-antihistamines decrease expression of ICAM-1 on cultured bronchial epithelial cells [176]. Zinc may also act as an antiviral agent by reducing ICAM-1 levels [177].

The monoclonal antibody to the cellular ICAM-1 was not effective. CFY196 (Coldsol) is a nasal spray multivalent Fab fusion proteins against ICAM-1 with a better avidity and * in vitro * potency against HRV [178]. Tremacamra, a soluble intercellular adhesion molecule 1 reduced the severity of experimental rhinovirus infection [179]. Pleconaril, an orally administered antiviral drug, acts by binding to a hydrophobic pocket in viral protein 1, and stabilizes the protein capsid so that the virus cannot release its RNA genome into the target cell. Outcomes of clinical trials with pleconaril have revealed mixed results and new compounds are currently being developed [180].

### Prevent RNA and protein synthesis

Despite extensive research, no agent has been approved for prevention and/or therapy of rhinovirus-induced diseases so far. Ruprintrivir selectively inhibits HRV 3C protease and shows potent, broad-spectrum anti-HRV activity * in vitro *. Ruprintrivir nasal spray (2% solution) prophylaxis reduced the proportion of subjects with positive viral culture by 26% and reduce viral titers, but did not decrease the frequency of colds [181]. HRV RNA synthesis during replication can be blocked by deoxyribozymes [182], morpholino oligomers [183], and small interfering ribonucleic acids [184]. The novel antiviral therapies that have been discovered recently, may one day add significantly to the armamentarium of antiviral agents, against respiratory viral infections in asthmatic children.

### Monoclonal antibodies

Maternally-derived RSV neutralizing antibodies help to protect infants against RSV hospitalization [185]. Palivizumab, a humanised monoclonal antibody against the RSV fusion protein is effective against RSV and wheezing in children and reduces hospitalization in high-risk individuals [185,187]. RSV prophylaxis with palivizumab significantly reduced the relative risk of subsequent recurrent wheezing in nonatopic premature infants [40]. Motavizumab is another monoclonal antibody against RSV, with an approximately 20-fold increase in ability to neutralize RSV and 100 fold increase in ability to reduce viral titers compared to palivizumab [188,189]. Motavizumab was also found to be superior to palivizumab in reducing outpatient medically attended lower respiratory illness by 50% [190].

### Vaccination

Vaccination against HRV and RSV have been in development for quite some time, but there are no safe and effective vaccines at present [33,191]. High rates of exposure to viruses in early life, presence of more than 100 serotypes of HRV, the presence of maternal antibodies, the risk of vaccine induced disease and relative immaturity of the infant immune system make effective vaccination difficult [186,192,193].

## Discussion

Respiratory viral infections are major contributors to the global burden imposed by asthma. In early life, they contribute to the inception of asthma and are responsible for most of the acute exacerbations for asthma in childhood. While the debate is not completely settled, children at high risk of developing asthma and those with established asthma may be at increased risk of acquiring respiratory viral infections and may be less able to contain these to the upper airway. Several simple general strategies can be used to help prevent respiratory viral infections in asthmatic children (Table 2), with good personal hygiene, hand-washing and avoidance of cigarette smoke likely to reduce respiratory viral infections. General immuno-stimulatory strategies, such as eating a healthy balanced diet, active probiotic supplements and bacterial-derived products, e.g. OM-85, may reduce recurrent infections in susceptible children.

**Table 2:** Summary of interventions to prevent rhinoviral infection in asthmatic children

**Effectiveness**	**Interventions**
**Most likely to be beneficial**	Hand Hygiene Immunostimulants (OM-85) Probiotics (specific strains), Prebiotics and Synbiotics Breast milk
**Likely to be beneficial**	Regular exercise, balanced diet, adequate sleep, low psychological stress Prevention of air pollutions and environmental tobacco smoke (ETS) Second generation of antihistamines Monoclonal antibodies: Anti IgE, Anti IL-5 Vitamin D, Vitamin A Garlic, zinc, ginseng Interferons
**Unknown effectiveness**	Montelukast Vitamin C Macrolides Echinacea Antiviral drugs
**Unlikely to be beneficial**	Mask Vaccination
**Likely to be ineffective or harmful**	Antibiotics Intensive exercise

## Summary

While research continues on specific anti-viral therapies, including vaccination, there are no currently available practical therapies that are suitable for widespread use. The role of preventative strategies in primary prevention of asthma in high risk children is of considerable academic interest and a number of studies are currently in the pipeline. The results are awaited with interest.

## Abbreviations

IFN: Interferon; PRRs: Pattern recognition receptors; TLRs: Toll-like receptors; HRV: Human rhinovirus; RSV: Respiratory syncytial virus; hMPV: Human meta-pneumovirus; LRI: Lower respiratory infections; sLRI: Severe lower respiratory infections; ETS: Environmental tobacco smoke; ICAM-1: Intercellular adhesion molecule-1.

## Competing interests

The authors declare they have no competing interests.

## Author’s contribution

HA and PDS conceived and designed the review. All authors reviewed the articles, abstracted data, and participated in the data synthesis. HA, PDS, YSC drafted the current manuscript, with critical review by PDS and CMJ. All authors contributed, read and approved the final manuscript.

## Pre-publication history

The pre-publication history for this paper can be accessed here:

http://www.biomedcentral.com/1471-2431/12/147/prepub
